# Superhydrophobic Polymer Composite Surfaces Developed via Photopolymerization

**DOI:** 10.1021/acsapm.1c00744

**Published:** 2021-08-19

**Authors:** Shreyas Pathreeker, Paul Chando, Fu-Hao Chen, Saeid Biria, Hansheng Li, Eric B. Finkelstein, Ian D. Hosein

**Affiliations:** † Department of Biomedical and Chemical Engineering, Syracuse University, Syracuse, New York 13244, United States; ‡ Syracuse Biomaterials Institute, Syracuse University, Syracuse, New York 13244, United States

**Keywords:** photopolymerization, superhydrophobicity, polymer composite, pattern, phase separation

## Abstract

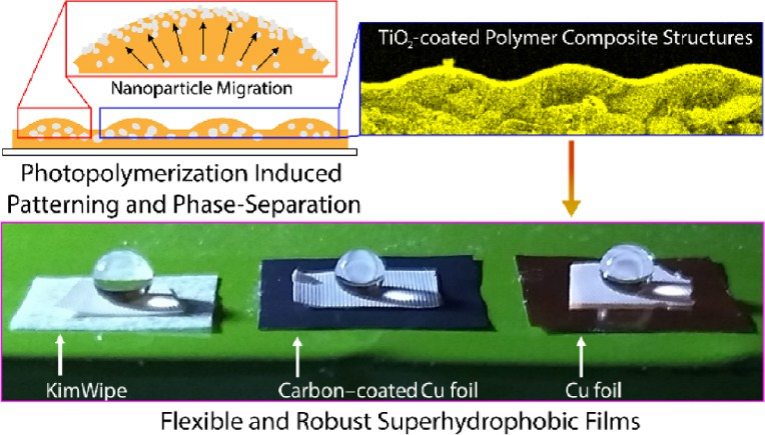

Fabrication of superhydrophobic materials using incumbent techniques involves several processing steps and is therefore either quite complex, not scalable, or often both. Here, the development of superhydrophobic surface-patterned polymer–TiO_2_ composite materials using a simple, single-step photopolymerization-based approach is reported. The synergistic combination of concurrent, periodic bump-like pattern formation created using irradiation through a photomask and photopolymerization-induced nanoparticle (NP) phase separation enables the development of surface textures with dual-scale roughness (micrometer-sized bumps and NPs) that demonstrate high water contact angles, low roll-off angles, and desirable postprocessability such as flexibility, peel-and-stick capability, and self-cleaning capability. The effect of nanoparticle concentration on surface porosity and consequently nonwetting properties is discussed. Large-area fabrication over an area of 20 cm^2^, which is important for practical applications, is also demonstrated. This work demonstrates the capability of polymerizable systems to aid in the organization of functional polymer–nanoparticle surface structures.

## 1 Introduction

Polymer materials with engineered surface functionality can be useful in the development of superhydrophobic surfaces. Fundamentally, “superhydrophobicity” is achieved when the angle of contact between a water droplet and the solid surface is >150°.^1^ Superhydrophobic materials typically consist of a combination of surface roughness on at least two different length scales, the micro- and nanoscales,^1,2^ and surface chemistries such as the use of fluorinated or silanized components,^3^ especially with polymers owing to their inherently nonhydrophobic nature. Due to the key role of surface roughness in antiwetting characteristics, imparting surface features like pillars,^4,5^ grooves,^6,7^ and Lotus leaflike papillae,^8^ or even random features using solvent evaporation,^9^ can enable the development of polymer or polymer composite [e.g., with nanoparticles (NPs)] materials with superhydrophobic behavior, especially for applications that place limitations on material weight yet require mechanical flexibility and robustness.^10^

Generally, developing such surface features can be easier with polymers than with inorganic materials like metals, which often require energy-intensive and/or complex techniques such as laser ablation, chemical etching, and lithography. This difference makes polymers an attractive material choice to develop textured or patterned superhydrophobic materials, both with^11^ and without NPs. Several techniques including, but not limited to, spray coating,^12−14^ solvent-assisted phase separation,^9^ and photopolymerization-based patterning^4,15^ have been reported in the literature to develop excellent polymeric superhydrophobic surfaces. Briefly, some of the limitations associated with these techniques (both for polymer and inorganic materials), spray coating requires several chemical components, including hydrophobic/surface-modified monomers and NPs, and the surface features are random or irregular.^12−14^ Mimicking a natural superhydrophobic surface on a metal (e.g., aluminum) substrate using a combination of photolithography, anodization, and replication consists of nine steps,^16^ whereas templating using metal meshes requires both the use of polytetrafluoroethylene (PTFE)^17,18^ and further processing that consists of three additional steps.^17^ The relatively simple dip coating of polymer–SiO_2_ composite formulations results in periodic but nonuniform-sized surface features.^19^ Etching and laser ablation can be used to develop patterned surfaces, but the former requires the use of toxic HF^20^ and the latter typically requires the use of fluorinated components.^21,22^ Simpler techniques like inkjet printing also require the use of fluorinated components.^23^ The development of both superhydrophobic pillars and superhydrophobic microporous surfaces using photopolymerization^4,15^ requires external deposition of a low-surface energy material such as PTFE particles, for example. Clearly, therefore, three key issues with incumbent techniques can be identified: (i) the use of fluorinated or silanized moieties, (ii) the involvement of multiple processing steps/lack of cost effectiveness, and (iii) external deposition of low-surface energy media onto the existing surface patterns, which may be construed as an additional processing step. Therefore, it is important to investigate and develop simpler and possibly more cost-effective methods to prepare superhydrophobic surfaces, as has been recently emphasized in the literature.^2^

Toward more spontaneous and less energy-intensive approaches to organize antiwetting polymer surface structures, conventional polymerization-induced phase separation (PIPS) has been used to develop superhydrophobic polymer surfaces. The process leverages the inherent immiscibility of formulation components to elicit the spontaneous organization of material structures with surface textures amenable to antiwetting properties. However, thus far, it has employed a solvent phase or a nonsolvent phase that facilitates random polymer phase separation,^9^ which in turn is expected to create sufficient surface roughness for water repellency, but often does not. Moreover, conventional PIPS offers little to no control over the regularity of the surface features, owing to the randomness of the spinodal decomposition. A succinct summary of the state of the art can be found in the literature.^1,2,10^

To address the challenges outlined above, here we report an approach based on photopolymerization-induced phase separation (referred to herein as PhIPS) in photopolymer–NP formulations to develop surface-patterned polymer composite superhydrophobic materials. PhIPS is a unique method for the directed organization of materials,^24^ which we leverage herein to uniquely organize structured materials simultaneously from two different components, that is, the polymer and the NPs without the use of solvents, in thin casted films. By inducing PhIPS in a casted thin film of the formulation, the photopolymer evolves into a substrate with periodically spaced “bumps” in the regions of elevated curing rate, and the phase separation of the NPs is directed to the top surface of the polymer substrate, thereby producing a dense, yet thin, NP overlayer. The result is a combined hierarchical surface structure as a result of two organization processes. Our samples display high water contact angles (WCA), low contact angle hysteresis (CAH), low roll-off angles (ROA), and excellent freestanding ability as well as flexibility while retaining their superhydrophobicity. The superhydrophobic property also enables our materials to display useful practical applications such as water repellency and self-cleaning. This work offers significant advantages over other incumbent techniques: developing superhydrophobic materials in a single processing step, without the need for fluorinated or silanized materials and without the use of external deposition of low surface energy materials. To the best of our knowledge, there is no report on developing superhydrophobic polymer composite materials combining surface patterning with concurrent PIPS of specific NPs to the surface of the polymer.

The novelty of our approach is that the coupling of photopolymerization-driven structural growth (i.e., periodically spaced microbumps) and simultaneous directed NP assembly leads to the generation of hierarchical surface roughness in a single step, wherein the synergistic effect of microscale patterns from the structures and nanoscale roughness from the NPs leads to exemplary superhydrophobicity. More specifically, PhIPS in a thin-film photopolymer–NP formulation directs NP migration toward the surface of the polymer during formation, thereby generating a uniform, conformal NP coating on top of the underlying polymer bumps. This contrasts with the general 3D, random phase separation expected in bulk photopolymer–NP media. This concurrent structure growth and spatial organization of NPs to the specific locations, that is, the surface of the polymer that is necessary to induce antiwetting characteristics, generate the surface features necessary for superhydrophobic properties. This work demonstrates the extraordinary capability of polymerizing systems to elicit dynamic processes to create functional material surface structures.

## 2 Experimental Section

### 2.1 Materials

Trimethylolpropane triacrylate (TMPTA) monomer and TiO_2_ NPs (nominal diameter ∼ 21 nm) were both purchased from Sigma-Aldrich, USA. Irgacure 784 photoinitiator was purchased from Ciba Specialty Chemicals, USA. All chemicals were used as received.

### 2.2 Preparation of Photopolymerizable Formulations

Photopolymerizable mixtures containing 3, 5, 8, and 16% TiO_2_ NPs (all w/w) were prepared by dispersing specific amounts of TiO_2_ NPs in TMPTA, followed by dissolving 1 wt % Irgacure 784 photoinitiator in the TMPTA–NP mixture. The final formulations were magnetically stirred for 24 h while being protected from ambient light. Prior to photopolymerization, all formulations were placed in an ultrasonic bath for 30 min in the dark to aid NP dispersion in the monomer matrix.

### 2.3 Photopolymerization

Photopolymerization was carried out by projecting 365 nm UV light (SOLIS-365C High-Power UV LED, Thorlabs, Inc.) through a chrome photomask (Photomask Sciences, Inc.) comprising a square array of 40 μm diameter circular apertures spaced 200 μm apart and into the photopolymerizable medium contained in Teflon ring cells (1 mm thick, 17 mm diam) glued to a plastic substrate on one end and the other end left open to the ambient air. Irradiation intensity for all samples was fixed at 650 mW/cm^2^, and irradiation times of 1, 4, and 24 h were investigated. Post irradiation, uncured resin was washed away using ethanol.

### 2.4 Materials Characterization

Scanning electron microscopy (SEM) and energy-dispersive spectroscopy (EDS) were carried out on a JEOL JSM-IT100LA and a JEOL JSM-5600 instrument, respectively. Imaging was performed using both secondary electrons and backscatter electrons at an accelerating voltage of 10 kV. High-magnification imaging was performed at 20 kV. Tapping-mode atomic force microscopy (AFM) imaging was carried out using a Veeco NanoScope IIIA instrument using a scan rate of 1 Hz.

### 2.5 Wettability Measurements

Static, advancing, and receding WCA measurements were carried out using a VCA Optima system (AST Products, Inc.). Advancing and receding measurements were collected by manually increasing or decreasing the volume of the water droplet, respectively, and the contact angles were measured by the system.

### 2.6 Flexibility and Self-Cleaning Measurements

Digital photographs and videos were captured using a 12 MP, f/1.8 aperture camera.

## 3 Results and Discussion

### 3.1 Concurrent Patterning and Phase Separation

**Figure 1:**
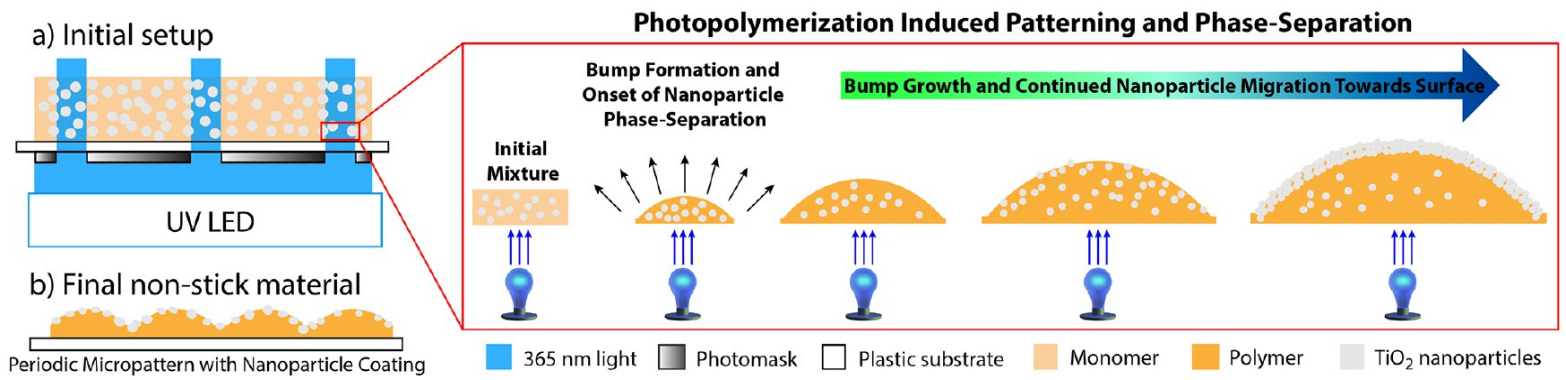
Schematic representation of the photopolymerization process used to develop micropatterned surfaces. Schematic (a) shows an initially homogeneous monomer–NP, which upon photopolymerization through a photomask leads to a patterned surface with micrometer-sized bumps, as shown in schematic (b). The magnified inset shows phase-separated NPs toward the surface of the bump-like structures, creating the nanoscale surface texture convolved with the large-scale pattern of the underlying polymer substrate.

Figure 1a illustrates a homogeneous monomer–NP mixture placed over a chrome photomask that was composed of 40 μm sized circular apertures spaced 200 μm apart. We used TiO_2_ NPs due to their versatility, low cost, and the ability to develop formulations that thermodynamically favor phase separation owing to the mismatch in solubility parameters between the NPs and the monomer. TMPTA was chosen for its fast polymerization kinetics. The photomask is used to generate individual micrometer-sized light beams, which generate a micropatterned polymer composite surface. Periodic photopolymerization is achieved by projecting UV light through the photomask and into the mixture, which leads to the development of a polymerized sample with bump-like surface textures centered in the regions of exposure, as shown schematically in Figure 1b. As the photopolymerization reaction proceeds, both the polymer chain length and molecular weight increase, thereby placing an entropic penalty on the NPs, owing to which the NPs seek less viscous, monomer-rich regions toward the top surface of the film and phase-separate outward. It is known that the radius of gyration *R*_g_ of the TMPTA monomer is 0.54 nm,^25^ and in this work, the radius of the TiO_2_ NPs, *R*_NP_, is ∼10 nm. Hence, as *R*_NP_ ≫ *R*_g_, the system is in the colloidal limit, and NP phase separation is expected.^25^

A wavy pattern with “peaks” and “troughs” is formed owing to periodic irradiation through the bright regions of the photomask, but as the incident light is scattered by the NPs, the dark regions not exposed to light also eventually undergo photocuring, forming a trough-like region between the bumps due to the lag between the polymerization between the bright and the dark regions. As a result, NPs that diffuse into the “dark regions” (i.e., nonirradiated) also undergo phase separation toward the surface, which was investigated and confirmed using EDS mapping (see Supporting Information). On average, across all weight fractions, these bumps are ∼65 μm tall. This process of concurrent organization of both the underlying polymer material and NP coating top layer is not only attractive as a straightforward approach to organize such hierarchical surface structures but also advantageous over NP deposition techniques, which can be energy-intensive and materially wasteful. The bump-like shape of the structures is due to the significant sideway scattering of light by the NPs, which is expected in NP-dense formulations. In systems without NPs, the same approach leads to pillar-like morphologies^4,26,27^ owing to a more forward propagation of light as opposed to sideways. Besides pattern generation, photopolymerization also induces phase separation of NPs toward the surface. This phenomenon has previously been reported using silicon NPs,^25^ semiconductor nanocrystals,^28^ silver decanoate,^29^ silica NPs,^30^ polymer blends,^31−33^ and polymer–solvent systems.^9,15^Figure 1b also shows a magnified view of a single bump, indicating the expected outward movement of NPs from the polymer-rich regions to monomer-rich regions (namely upward to the surface) owing to PIPS, which is key to good surface coverage.

### 3.2 Materials Characterization

**Figure 2:**
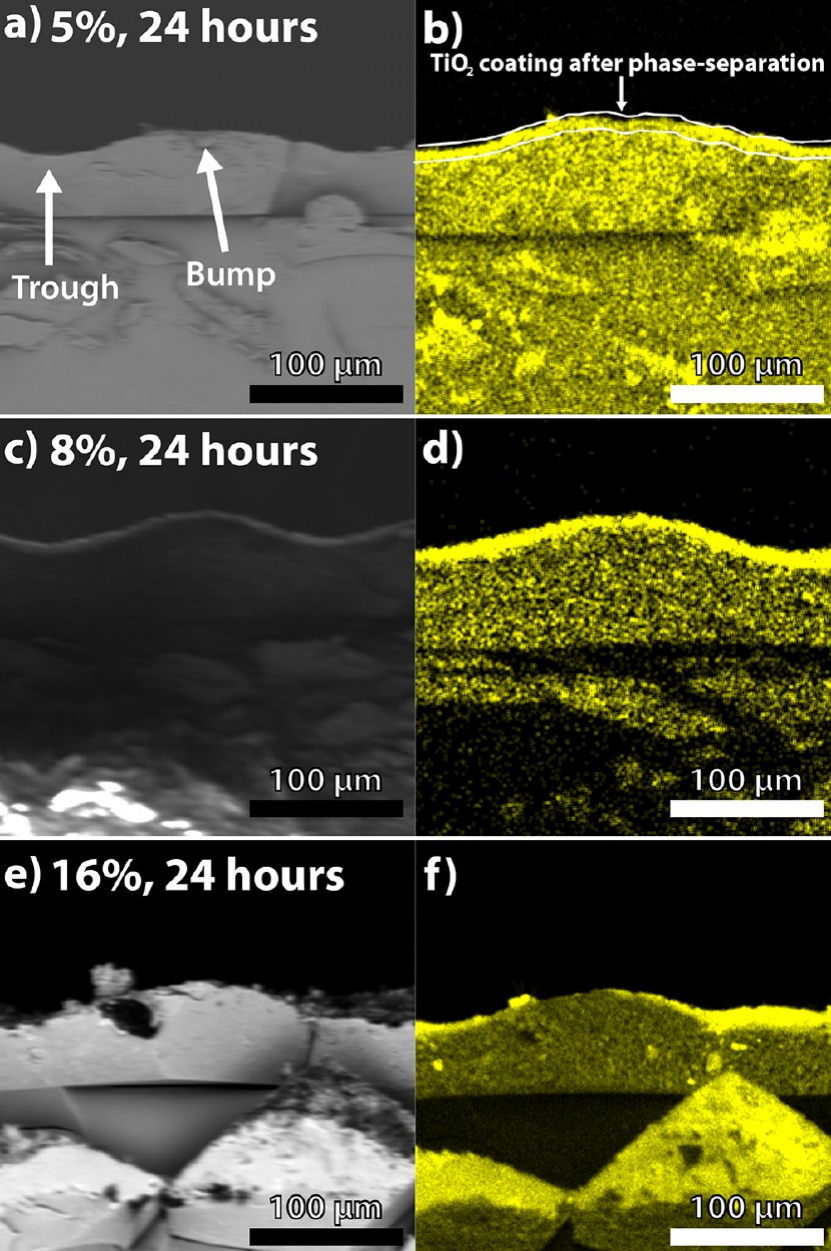
Cross-sectional SEM images and EDS maps for titanium shown for samples containing (a,b) 5%, (c,d) 8%, and (e,f) 16% NPs, all obtained with an irradiation time of 24 h.

Owing to the critical role of NP surface coverage and surface roughness in superhydrophobicity, our first objective was to confirm photopolymerization-induced NP phase separation at the individual bump level. Figure 2 shows the backscatter SEM micrographs and EDS maps for titanium (representative of TiO_2_) for the cross sections of individual bumps in samples containing 5, 8, and 16% TiO_2_ obtained after an irradiation period of 24 h. Results for 3% samples are shown in the Supporting Information as they only displayed hydrophobicity but not superhydrophobicity. The SEM images shown in Figure 2a,c,e confirm that the structures were sliced through cross sections, thereby eliminating the possibility of EDS mapping on the top surface as opposed to the bump cross section, which could result in inaccurate maps.

The EDS maps shown in Figure 2b,d,f confirm that the top surfaces of the bumps in all samples comprise a TiO_2_ coating of varying thicknesses, indicating successful NP phase separation and surface coverage. Relatively better NP phase separation and uniform surface coating were observed for samples containing 8% NPs (Figure 2d), whereas irregular surface coverage was observed for samples containing 16% NPs (Figure 2f), which we attribute to excess NP aggregation and bulk agglomeration owing to excessive NP loading. In the case of 5% NP loading, a more uniform distribution of NPs across the entire bump was observed, which is likely owing to the relatively lower NP loading. Nonetheless, these images confirm that PhIPS of NPs occurs across all NP weight fractions. Similar NP coatings were found across all irradiation times explored as well (Figures S1–S4, Supporting Information).

Across all weight fractions, no significant change in bump height was observed with the increase in irradiation time, which is likely due to the attainment of a steady state in the structure growth after ∼1 h of irradiation. Such behavior has previously been reported in NP–TMPTA^25^ and pure TMPTA^26^ systems owing to the consumption of free radicals, which slows down polymerization. However, the system is in a weakly cross-linked, sol–gel-type state wherein NP diffusion can still occur.^25,32^ These results confirm the presence of NPs on the surface, and consequently, nanoscale roughness, which is necessary for imparting superhydrophobic properties to the otherwise hydrophilic polymer.

**Figure 3:**
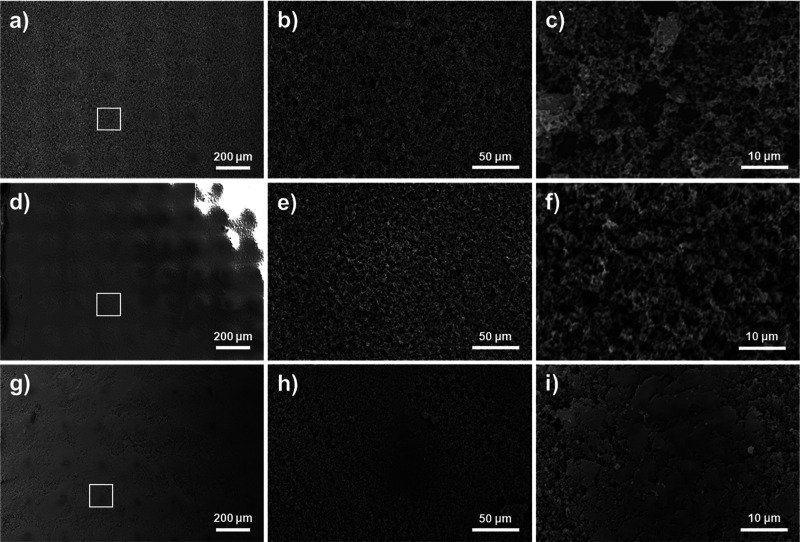
Top-down SEM images for photopolymerized samples containing (a-c) 5% (d-f) 8%, and (g-i) 16% NPs, all obtained after irradiation for 24 h. Insets show the top surfaces of the corresponding individual bumps.

Top-down scanning electron micrographs of the surfaces of photopolymerized samples containing 5, 8, and 16% NPs obtained after 24 h of irradiation are shown in Figure 3. Both the uniformly distributed bump-like structures and the overall surface roughness are clearly visible for all weight fractions, as well as all irradiation times (Figures S5–S8, Supporting Information), thereby confirming the microscale surface roughness of our samples and the ability to create such structures in NP-dense formulations. The top-down EDS mapping of an 8%, 24 h sample also reveals surface coverage with TiO_2_ NPs (Figure S9, Supporting Information). The SEM images in the cross sections shown in the Supporting Information more clearly reveal the structure of the bumps.

The spacing between the structures is 200 μm, the same as the spacing between the apertures of the photomask used herein. Higher magnification insets reveal the microscale and submicron-scale roughness of the surface of the bumps as well as the highly porous nature of the surfaces. These micrographs confirm the hierarchical roughness of our sample surfaces, which is observed for all samples (see Supporting Information). Trapping of air in pores is essential to achieve superhydrophobicity (Cassie–Baxter wetting^34^). Therefore, although surface roughness is evident for all samples, surface porosity was visually found to be higher for samples containing 5% NPs (Figure 3c) and 8% NPs (Figure 3f) over all irradiation times as compared to those containing 16% NPs (Figure 3i).

Representative AFM height images of samples cured for 24 h are shown in Figure S10 (Supporting Information). Mean surface roughnesses observed were between 218 and 323 nm. These values agree with the submicron-sized features observed in the SEM images. Therefore, collectively, the SEM and AFM images reveal the micro- and nanoscale surface roughnesses of our samples, which in combination with the NP coating observed in the EDS maps shown in Figure 2 are expected to result in the antiwetting behavior. Surface composition characterization of an 8%, 24 h sample using Fourier transform infrared (FTIR) analysis revealed the composite nature of the sample surface consisting of both TiO_2_ and TMPTA (see Figure S11 and Table S1, Supporting Information). This presence of TMPTA provides the necessary adhesion of the NPs to the underlying polymer film, the importance of which will be discussed later. High advancing contact angle (ACA), high receding contact angle (RCA), and low CAH are the key markers used to assess the superhydrophobic surface behavior.^2^

### 3.3 Surface Wettability Characterization

**Figure 4:**
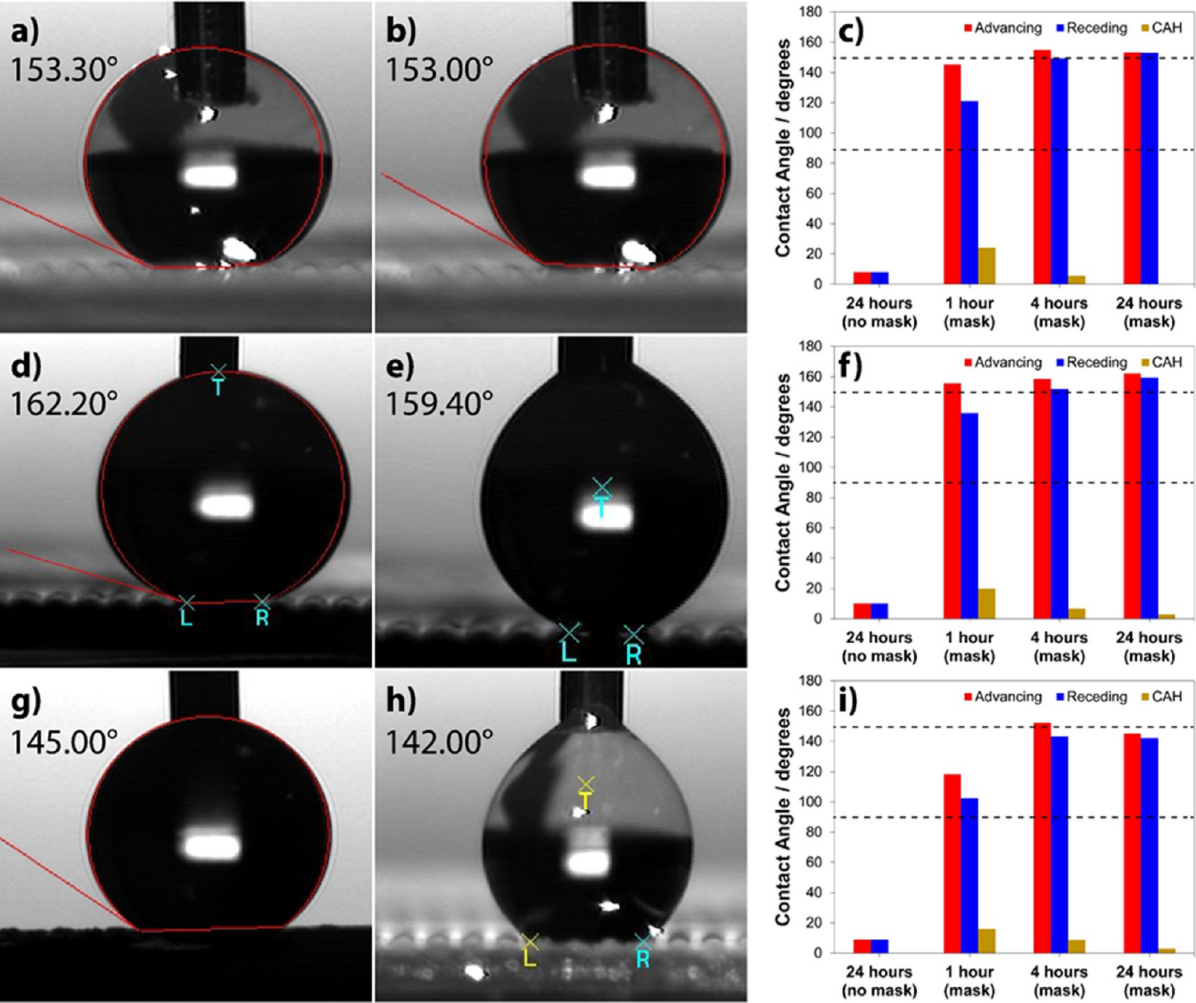
Contact angle goniometry images taken during advancing and receding measurements for samples containing (a,b) 5%, (d,e) 8%, and (g,h) 16% NPs, all obtained after an irradiation time of 24 h. Bar plots summarizing advancing and RCA values as well as CAH values for samples obtained at different irradiation times for formulations containing (c) 5%, (f) 8%, and (i) 16% NPs. Dashed lines indicate the hydrophobic limit (90°) and the superhydrophobic limit (150°).

Contact angle goniometry images during advancing and receding measurements for samples containing 5, 8, and 16% NPs are shown in Figure 4. ACA, RCA, and CAH measurements are summarized in the bar plots for structures produced with NP weight fractions and increasing irradiation time. The specific numerical values are provided in the Supporting Information.

To investigate the possibility of a critical NP concentration required to achieve superhydrophobicity, we also synthesized samples containing 3% NPs using the same irradiation times of 1, 4, and 24 h (EDS and SEM images for these can be found in the Supporting Information). ACA, RCA, and CAH values for all weight fractions and all irradiation times are also summarized in Table S2, Supporting Information. Notably, in accordance with the observations from the SEM analysis, the highest ACA and RCA values were observed for samples containing 5 and 8% NPs owing to the similarity in their surface roughnesses and bump morphologies.

Comparative contact angle values for samples cured for 24 h, but without the use of a photomask (i.e., uniform exposure), are also shown in the bar plots (Figure 4c,f,i) to highlight the differences in ACA and RCA values between the patterned and nonpatterned surfaces. The WCA values for these “no mask” samples are ∼10° for all weight fractions (see Figure S12, Supporting Information), which clearly reveal the critical nature of a patterned surface topology in imparting antiwetting properties, with surface structures as short as 65 μm being sufficient to impart superhydrophobicity to the material. This comparison also confirms that the synergistic combination of pattern- and NP-imparted surface roughness and porosity results in the superhydrophobic behavior. Samples containing 3% NPs demonstrated hydrophobicity (WCA ∼ 120°, Figure S13, Supporting Information), most likely owing to lesser NP content and lesser surface roughness, as seen in the SEM images, but not superhydrophobicity, which indicates that a NP concentration of at least 5% is necessary to develop superhydrophobic polymer composites. Excessive NP weight fractions (≥16%) are detrimental to the overall superhydrophobicity, owing to the coverage of the underlying bump pattern and the bulky nature of the coating (see Figure S14b, Supporting Information).

Overall, the comparison of the contact angle results for all irradiation times (Figures S15–S17, Supporting Information, for 5, 8, and 16% NP loading, 1 and 4 h irradiation times) reveals that for all weight fractions, both ACA and RCA values increase slightly with the increase in the irradiation time, with samples containing 5% NPs (4 and 24 h irradiation) and 8% NPs (4 and 24 h irradiation) demonstrating contact angles exceeding the superhydrophobic limit. For samples containing 16% NPs, the highest contact angles are obtained after 4 h of curing instead of 24 h. Furthermore, CAH values decrease with the increase in irradiation time across all weight fractions. For all weight fractions, the average ACA difference between the nonpatterned and patterned surfaces was found to be ∼140°, pointing toward the critical nature of surface topology (both the polymer structure and NP overlayer) in imparting antiwetting properties. To further highlight the importance of the combination of NPs and periodic patterns, we fabricated a uniform film of TMPTA (no photomask) and pure TMPTA pillars (using the same photomask dimensions) and found static WCA values of <90° (Figure S18, Supporting Information). Although both irradiation times of 4 and 24 h facilitate the development of superhydrophobic or near-superhydrophobic surfaces with samples containing 5 and 8% NPs, an irradiation time of even 1 h leads to the superhydrophobic behavior but with the limitation that the RCA value would be lower than 150°. Between the samples containing 5 and 8% NPs, the highest ACA, highest RCA, and low CAH values were obtained with 8% NP loading and 24 h irradiation time. While the 5%, 24 h sample displayed the lowest CAH value, its ACA and RCA values were lower than that for its 8% counterpart. Therefore, the 8%, 4 h and 8%, 24 h samples were chosen for further characterization and analysis. Summarily, it appears based on our data that 5% NP loading accompanied by surface patterning was sufficient to impart superhydrophobic properties to the otherwise hydrophilic TMPTA monomer, but 16% weight loading resulted in near-superhydrophobic contact angle values. These results indicate the importance of NP content on surface properties, namely higher NP content leads to higher contact angle values, but excessive nanoparticle coating is detrimental. Lastly, it must be noted that no surface modification of the nanoparticles or monomers (such as silanization or fluorination) was carried out herein and that our superhydrophobic samples are fabricated in a single step (excluding the washing step).

### 3.4 Postprocessability

**Figure 5:**
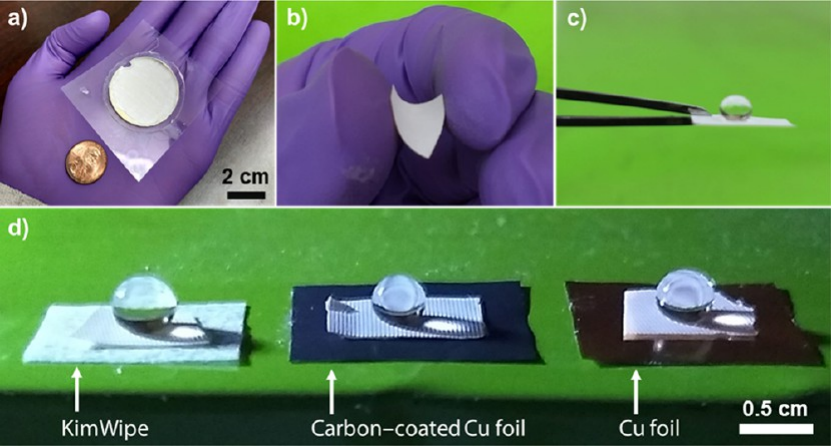
Digital photographs showing (a) exemplary postprocessability of an 8%, 4 h sample obtained by photopolymerization over a 20 cm^2^ area, (b) same sample peeled off and bent to demonstrate flexibility, (c) freestanding ability and retention of superhydrophobicity shown using a sessile water droplet, and (d) a section of the same sample peeled off and attached to different substrates.

Superhydrophobic coatings are typically applied to other substrates such as cloth and metal for practical applications, requiring that the coatings be easy to process. It is also important that such materials be developed in a scalable manner to cover large surfaces such as fabrics and metal sheets exposed to the elements. The capability of our samples in these aspects is shown in Figure 5. The possibility of relatively large area synthesis using our approach is shown in Figure 5a. The sample shown herein was obtained by photopolymerization of a resin containing 8% NPs using an exposure time of 4 h (ACA, 158°and RCA, 152°) in a 5 cm diameter ring cell on a commercial plastic sheet. This led to a superhydrophobic sample with a 20 cm^2^ surface area (video of a water droplet rolling on this surface can be found in Movie S1, Supporting Information). A standard US penny, which measures ∼19 mm (or 0.75″) in diameter, is also shown along with the sample for the purpose of visual size comparison. It was also possible to easily cut our sample using a razor blade, peel it off, and bend it without any breakage or damage, as shown in Figure 5b, which demonstrates the robustness of the bump-like structures and the overall flexibility of our sample owing to its elastomeric nature.

The thin film’s retention of superhydrophobicity after bending is shown in Figure 5c, wherein the same freestanding film displays exemplary nonwettability, indicating a robust NP coating on the surface and preservation of the underlying surface texture after processing. Albeit our approach here is not a direct coating technology, we attempted to use the peel-and-stick technique with our sample to demonstrate its ability to be placed on different substrates, while it retained its superhydrophobicity. In this work, peel-and-stick refers to attaching the sample to other substrates using commercially available Scotch tape. These results are shown in Figure 5d, wherein a section of our sample was attached to a piece of KimWipe, a piece of carbon-coated copper foil, and a piece of copper foil each. Sessile water droplets placed on these samples remain intact without wetting the surface. The underlying surface pattern of our samples is also clearly visible. While the sample on all underlying substrates is the same, these results demonstrate the exemplary retention of antiwetting characteristics after the processing maneuvers such as peeling, bending, and pressing. Overall, our results indicate that the sample is mechanically robust yet flexible so as to withstand the postprocessing maneuvers without losing its antiwetting property. However, it must be noted that sample handling must be carried out carefully so as to not damage the surface structures (bumps).

### 3.5 Water Repellency

**Figure 6:**
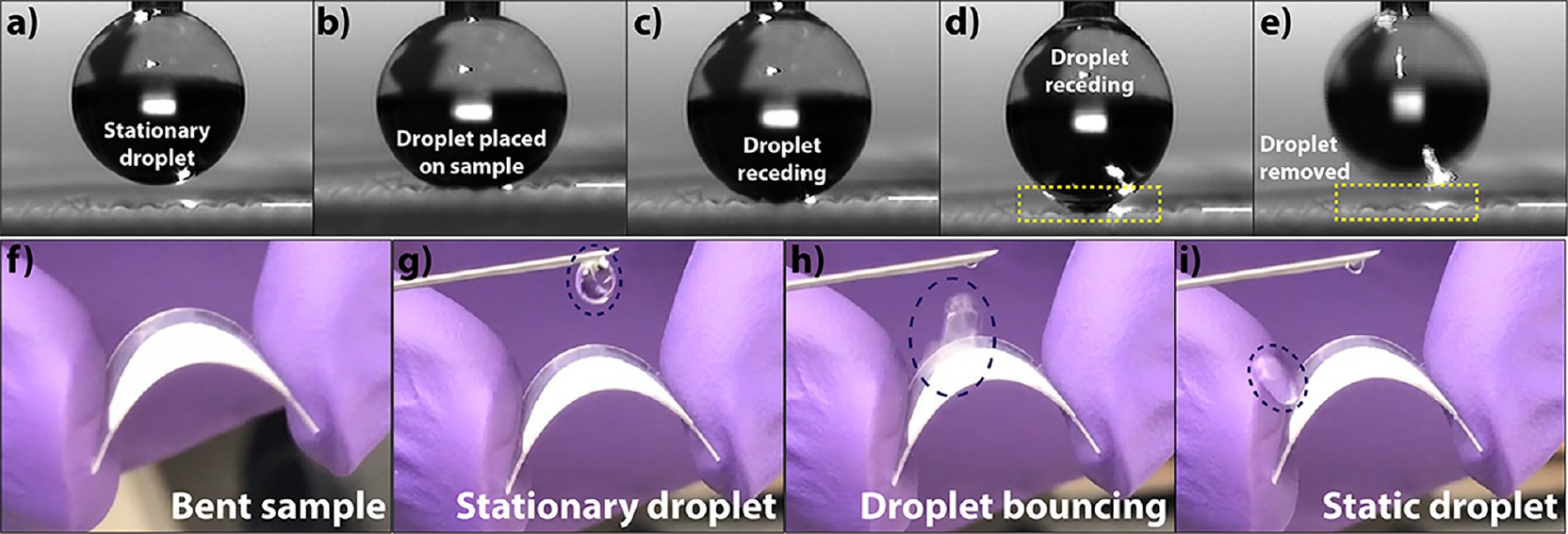
Digital photographs (a−e) showing the nonstick nature of an 8%, 24 h sample by placing and removing a sessile water droplet on the surface of the sample. Yellow boxes in panels (d,e) indicate the region of contact between the droplet and the surface of the sample where no residue of water is found after droplet removal. Similarly, panels (f−i) show a curved 8%, 24 h sample with a water droplet bouncing off the surface of the sample and sticking to a rubber glove, as shown in panel (i), indicating exemplary nonwetting characteristics of the sample.

Figure 6 shows snapshots from a dynamic video capture of a water droplet being placed onto and removed from the surface of an 8%, 24 h sample.

A stationary water droplet is shown in Figure 6a, followed by its placement on the surface, as shown in Figure 6b, wherein the droplet is placed onto the substrate and its volume slightly increased. The stage is also elevated slightly at this point to gently compress the water droplet. Removal of the droplet by lowering the stage commences in Figure 6c and continues in Figure 6d, wherein the droplet is retracted from the surface of the sample, with the dashed box in Figure 6d indicating the diminishing area of contact between the water droplet and our sample, and Figure 6e finally shows the droplet completely intact leaving no trace of water on the sample surface. No droplet spreading was observed on the surface of the sample even after compressing the droplet (Figure 6b), which indicates the ability of our sample to repel water owing to the high ACA and RCA values. Often during practical use, water repellency is required even when the surface is bent or curved. Bouncing of water droplets off superhydrophobic surfaces is governed by several factors such as the height from which a droplet strikes the material surface, angle of impingement, and volume of the droplet, among others. A surface with rounded microstructure displaying a contact angle >151° can be expected to demonstrate water droplet bouncing if it is indeed superhydrophobic.^35^ As the microstructure of our samples and their high ACA and RCA values (in this case, ACA of 162° and RCA of 159°) satisfy these conditions, we further attempted to demonstrate the water repellency capability of our sample. The sample was bent while it was still on the plastic substrate to create a curved surface, as shown in Figure 6f, and a water droplet was placed on it to assess its antiwetting property. Figure 6g shows the water droplet just prior to being suspended onto the bent substrate. This water droplet in Figure 6h can be seen bouncing off the curved surface of our sample and sticking to the hydrophilic rubber glove, highlighted by the dashed oval in Figure 6i. Full video of flexibility can be found in Movie S2, Supporting Information, wherein several water droplets were impinged onto the curved surface from different heights.

Low ROA values are also representative of the Cassie–Baxter-type superhydrophobic behavior of a surface.^2^ Our most optimum sample (8%, 24 h) displayed a ROA of 1°, with two water droplets suspended onto the surface from different heights (Movie S3, Supporting Information), and traceless nonstick water droplet movement (Movie S4, Supporting Information), corroborating the high ACA, high RCA, and low CAH values seen in Figure 4f. These results highlight the fact that combining surface patterning and NP coating confers exciting functionalities to the superhydrophobic surface. Lastly, again in the context of practical use of our material, we investigated the so-called self-cleaning ability of our optimum sample (8%, 24 h) using graphite shavings placed on the surface of the sample.

### 3.6 Self-Cleaning Ability

**Figure 7:**
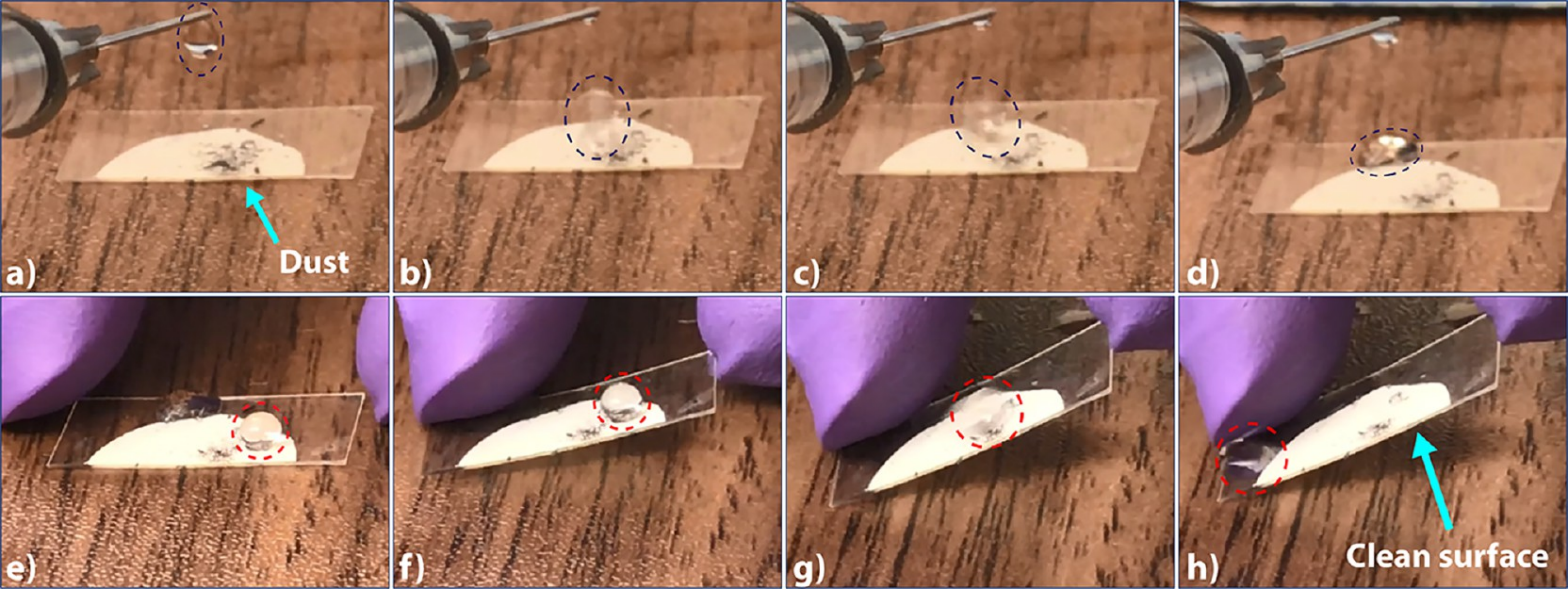
Digital photographs showing the self-cleaning ability of an 8%, 24 h sample. Panel (a) shows a dust-covered surface just prior to being cleaned by suspending a water droplet onto it. Panels (b−d) indicate the water droplet bouncing onto and away from the surface of the sample and toward the less hydrophobic plastic substrate after collecting the dust from the surface. Panel (e) shows a sessile water droplet placed on the same surface, whereas panel (f−h) shows the sample being tilted to roll the droplet, which also leads to a clean surface.

Figure 7 shows the digital photographs of graphite dust placed on the surface of an 8%, 24 h sample which itself is placed on a flat table (full video can be found in Movie S5, Supporting Information). The dashed oval in Figure 7a shows the water droplet just before being suspended onto the sample. In Figure 7b, the droplet bounces off the surface and travels to the highly hydrophilic plastic substrate (Figure 7c. The final, stationary droplet along with the collected dust and the clean region of the initially dusty surface are all clearly visible in Figure 7d. The sample was then lifted off the edge, as shown in Figure 7f, to allow the water droplet to roll off, which can be seen in Figure 7g, clearly outlined by the dashed red oval.

The droplet rolled off and collected all the dust in its pathway, with both the final droplet and clean surface shown in Figure 7h. In this test, a higher ROA (∼5°) was necessary to achieve droplet movement and sliding owing to the effect of gravity on the initially stationary water droplet. Nonetheless, this particular ROA value also falls within the acceptable range for superhydrophobic materials. The results shown here, namely high ACA, high RCA, low CAH, and low ROA, can be attributed to successful patterning and NP phase separation, with the FTIR results confirming NP–monomer adhesion, which aids in the retention of superhydrophobicity during and after the postprocessing steps. Our findings are also representative of other advanced superhydrophobic materials.^36^ Additional digital photographs showing the exemplary antiwetting property of our samples (such as multiple droplets on the same sample) can be found in Figure S19, Supporting Information.

### 3.7 Mechanical Durability

**Figure 8:**
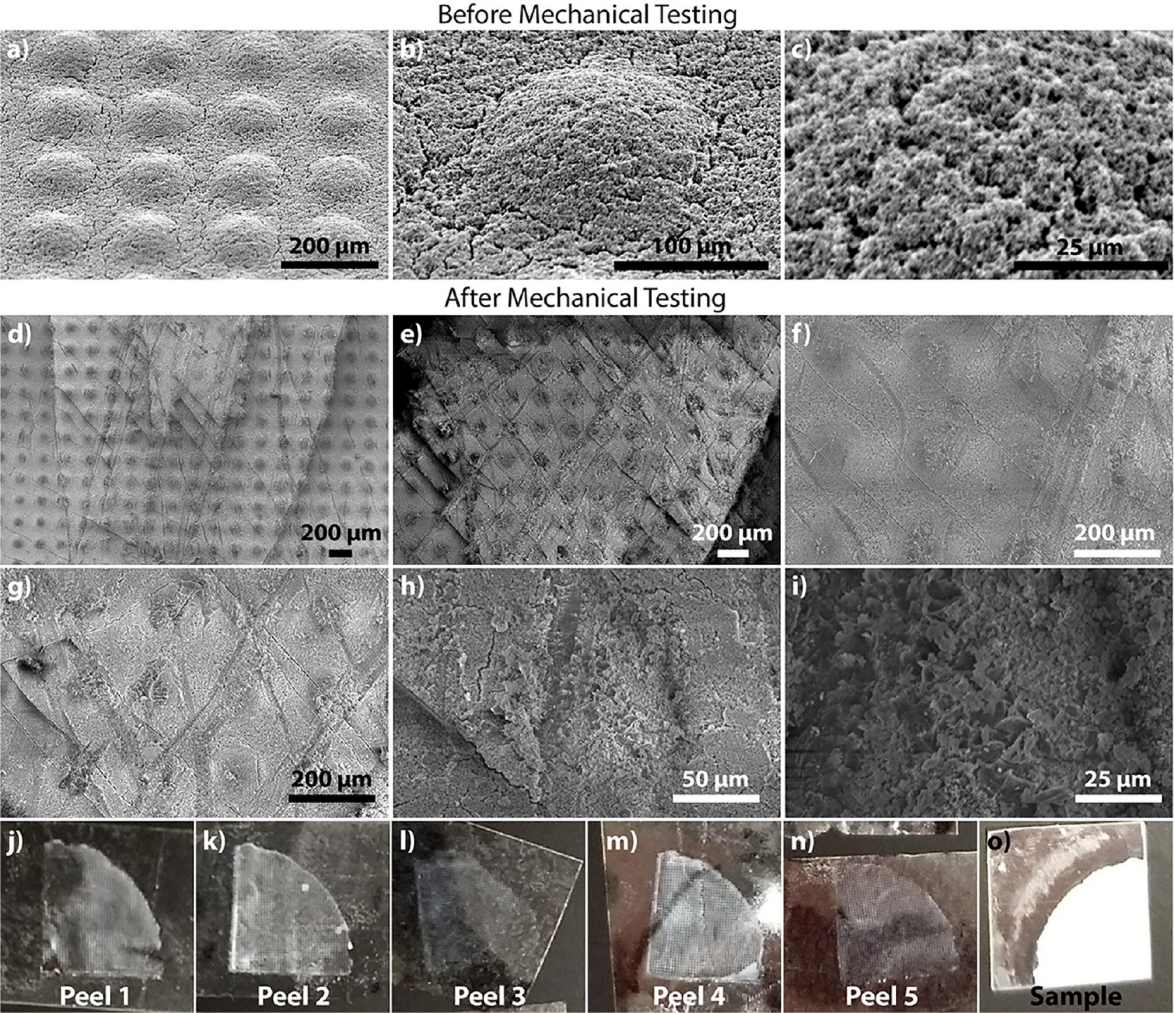
SEM images for an 8%, 24 h sample (a–c) before mechanical testing and (d–i) after mechanical testing, which consisted of five consecutive Scotch tape peels, followed by a first abrasion with sandpaper, then placing the abraded sample under a stream of tap water, and finally by a second, final abrasion with sand–paper. Damage to the surface of the sample is clearly visible. Panels (j–n) show digital photographs of the layers peeled off, whereas panel (o) shows the sample after peel-off tests.

The mechanical durability of our sample was assessed using the Scotch tape method (see Movie S6, Supporting Information) and the sandpaper abrasion method (see Movie S7, Supporting Information) (both performed consecutively on the same sample section). The SEM images of this sample before and after testing are shown in Figure 8, along with the digital photographs of the peeled off pieces of the tape, and the sample after peel-off. Figure 8a–c shows the sample before mechanical testing, wherein the micrometer-sized bumps are clearly visible. The surface roughness of these bumps can also be seen clearly. Figure 8d–i shows the sample after mechanical testing, which consisted of the following steps: (1) five consecutive Scotch tape peel-offs, (2) sandpaper abrasion, (3) placing the sample under a stream of tap water, and (4) a second sandpaper abrasion. Damages to the sample in the form of several cracks, streaks, and abrasion are clearly visible. From the five peel-off tests [panels (j–n)], it appears that some NPs come off the surface (white residue on the tape) in decreasing amounts. After the completion of mechanical testing, however, the underlying polymer surface pattern remains intact, which may be attributed to the strongly cross-linked polymeric structure owing to an extended (24 h) exposure time. It also appears that the two abrasion maneuvers (consisting of four rubs each) flatten the bumps to a certain extent, as a result of which the surface roughness slightly decreases. Nevertheless, the sample surface still appears to be quite rough even after mechanical testing, which, when combined with the NPs (see EDS maps in Figure 2) in the sample across the *z*-direction, aids the retention of antiwetting behavior. These characteristics are beneficial as they convey mechanical robustness and, as a result, retention of superhydrophobic or near-superhydrophobic surface properties even after significant wear and tear to the polymer composite.

**Figure 9:**
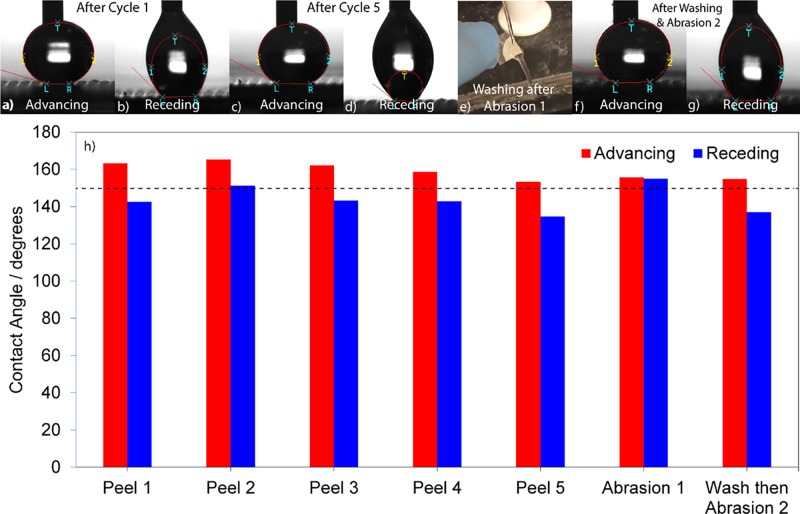
ACA and RCA measurements of an 8%, 24 h sample after (a,b) first peel-off, (c,d) fifth peel-off, and (f,g) after all the mechanical tests were completed. Panel (e) shows the sample under a stream of tap water after the first abrasion. (h) Bar plot summarizing the ACA and RCA values of the sample over the course of mechanical testing.

Contact angle measurements of the sample after each mechanical test as well as representative advancing and RCA goniometry images are shown in Figure 9. ACA values after the first three peel-off tests approach 163° and then decrease to ∼153°, bringing the average ACA after tape peeling to 159°. RCA values on average remain ∼143°, which then lead to an average CAH value of 16°. In comparison to ACA, RCA, and CAH values before testing (see Figure 4), we attribute the high CAH value to the decrease in the overall NP coverage, as evidenced by the images shown in Figure 8.

Yet, the preservation of the antiwetting behavior is clearly evident from these values owing to the preservation of the polymer and its pattern. After abrasion 1, the sample displays ACA and RCA values ∼152°, with a low CAH value, which may be attributed to the underlying NPs being exposed after the abrasion, which, in comparison with the surface NPs, could possess lesser hydroxyl groups. As for the retention of the superhydrophobic behavior after abrasion, the SEM images shown in Figure 8 clearly reveal the prevailing surface roughness. Finally, after washing the sample with water (see Movie S8, Supporting Information) and performing a second abrasion test, it was observed that although the ACA value was ∼152°, the RCA value was ∼137°, indicating high CAH, which we attribute to the further reduction in surface roughness caused by significant abrasion. Based on the increase in the CAH value from 3° before mechanical testing to ∼15° after mechanical testing, it appears that the sample, owing to the partial degradation of the surface structures, undergoes a transition from the Cassie–Baxter wetting state to the Wenzel wetting state, still retaining its overall antiwetting behavior, as evidenced by the contact angle values shown in Figure 9. Decrease in RCA values as well as the Cassie–Baxter to Wenzel transition owing to abrasion has recently been reported in the literature for polystyrene NP surfaces, wherein recovery of superhydrophobicity was achieved by repetitive spray coating of the NPs.^11^ In our case, exposing the underlying surfaces after abrasion also appears to result in an analogous result, wherein the superhydrophobicity of the sample is retained. ACA and RCA goniometry images after different mechanical testing steps (Figure S20), as well as additional movies of contact angle measurements after peel 1 (see Movie S9), after peel 5 (see Movie S10), after abrasion 1 (see Movie S11), and after abrasion 2 (see Movie S12), are available in the Supporting Information.

## 4 Conclusions

In summary, we have demonstrated that the simultaneous combination of photopolymerization-induced patterning and NP phase separation is a simple, scalable, and effective approach for the development of textured materials, with the hydrophobic and superhydrophobic surface properties demonstrating WCAs in the range of 120–162°. The materials fabricated using our technique are mechanically robust yet flexible and demonstrate exciting properties such as water repellency and self-cleaning owing to their excellent superhydrophobicity.

While we are actively pursuing top-down curing, the approach reported here can be used to fabricate superhydrophobic materials that can be freestanding and attached to other substrates, while retaining their superhydrophobicity during and after handling postprocessing steps. The fact that the sample can be peeled off indicates that a transparent substrate such as glass or plastic is not a necessity, and future work is aimed at developing such materials more rapidly using a combination of lower resin thickness and higher light intensity, which would also allow these materials to be more transparent owing to lesser scattering of light across the film. Other key focus areas are to employ our approach in developing superomniphobic materials by the suitable choice of monomers and NPs (either nonfunctionalized or functionalized) and to explore the fabrication of such materials using pretextured substrates, so as to eliminate the use of a photomask.

## Supporting Information Available

Backscatter SEM images and EDS elemental maps in cross sections for 3, 5, 8, and 16% samples (1, 4, and 24 h); SEM images in top-down mode for 3% (1, 4, and 24 h), 5, 8, and 16% samples (1 and 4 h); EDS map in top-down mode for 8% (1 and 4 h); AFM images for 5, 8, and 16% samples (24 h); FTIR spectrum of 8%, 24 h sample and the corresponding peak assignments; summary of ACA, RCA, and CAH values; static contact angle images for “no mask” samples; contact angle images for 3% samples (1 and 4 h); digital photographs of perspective view and water droplet on cracked surface; contact angle images for 5, 8, and 16% samples (1 and 4 h); contact angle images for pure TMPTA samples; additional digital photographs of sessile water droplets in various configurations; and contact angle images for an 8%, 24 h sample after different mechanical testing steps (PDF)
Droplet rolling on a large sample (MP4)
Droplet bouncing on flexible surface (MP4)
ROA (MP4)
Drag-free droplet movement (MP4)
Self-cleaning (MP4)
Scotch tape peel-off MP4)
Sandpaper abrasion (MP4)
Water wash step (MP4)
Contact angle measurement after peel 1 (MP4)
Contact angle measurement after peel 5 (MP4)
Contact angle measurement after abrasion 1 (MP4)
Contact angle measurement after abrasion 2 (MP4)
