# Supplementary material for: Superhydrophobic Polymer Composite Surfaces Developed via Photopolymerization
Source: ACS Appl Polym Mater. 2021 Aug 19;3(9):4661–72. doi: 10.1021/acsapm.1c00744 (PMC8438665; doi:10.1021/acsapm.1c00744)
Supplement: Supplementary file 1 — ap1c00744_si_001.pdf [file ap1c00744_si_001.pdf]

Supporting Information

Superhydrophobic Polymer Composite Surfaces Developed via Photopolymerization

Shreyas Pathreeker[a], *Paul Chando*[a], *Fu–Hao Chen*[a], *Saeid Biria*[a], *Hansheng Li*[a], *Eric B. Finkelstein*[a, b], *and Ian D. Hosein*[a]*

[a]Department of Biomedical and Chemical Engineering, Syracuse University, Syracuse, NY 13244, United States

[b]Syracuse Biomaterials Institute, Syracuse University, Syracuse, NY 13244, United States

*Corresponding author: Prof. Ian D. Hosein, idhosein@syr.edu

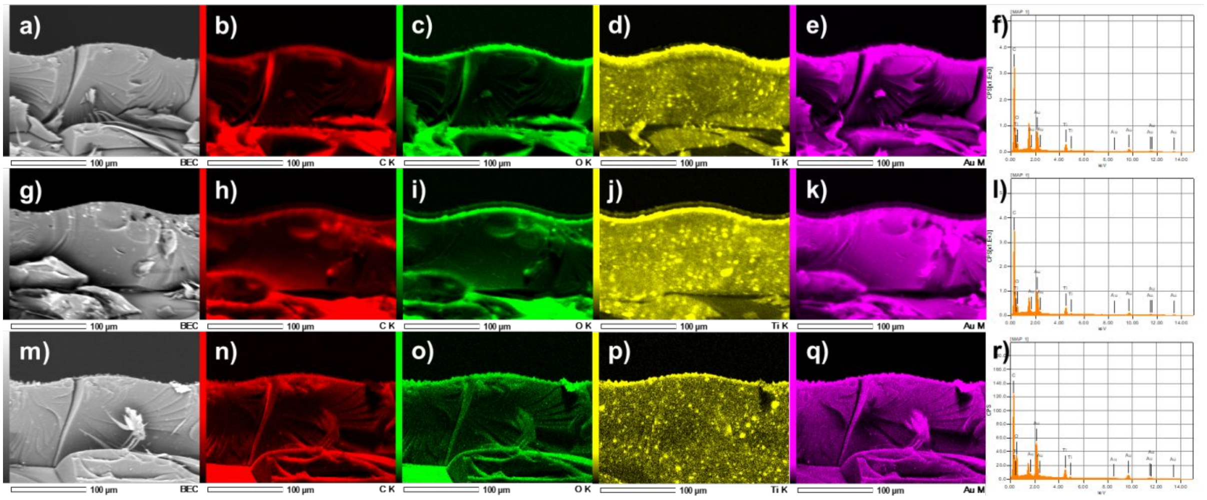

Figure S1. SEM images and EDS maps in cross–section of samples containing 3% nanoparticles obtained after exposure times of a)–f) 1 hour, g)–l) 4 hours, and m)–n) 24 hours. Corresponding spectra are also shown.

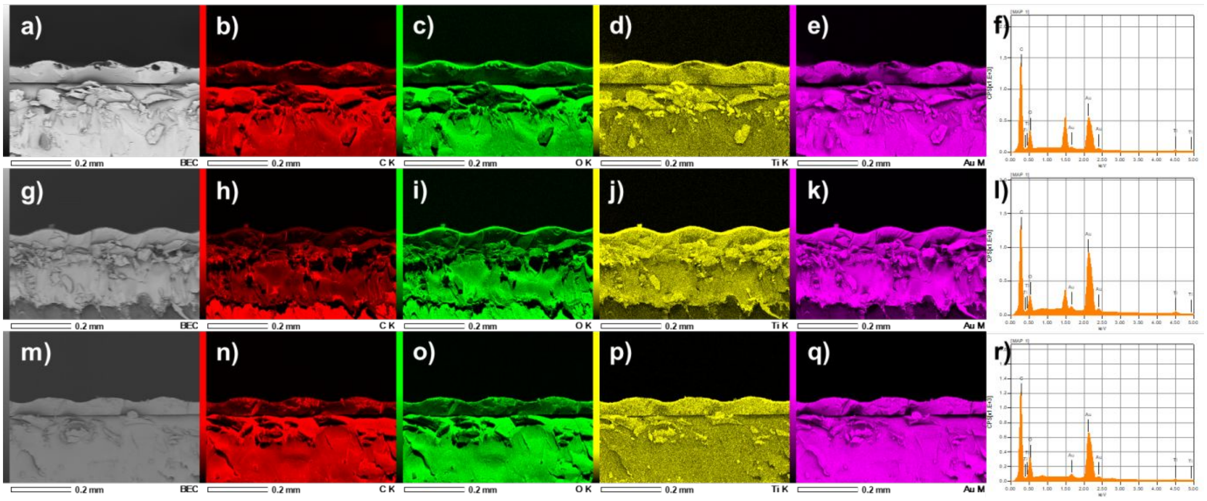

Figure S2. SEM images and EDS maps in cross–section of samples containing 5% nanoparticles obtained after exposure times of a)–f) 1 hour, g)–l) 4 hours, and m)–n) 24 hours. Corresponding spectra are also shown.

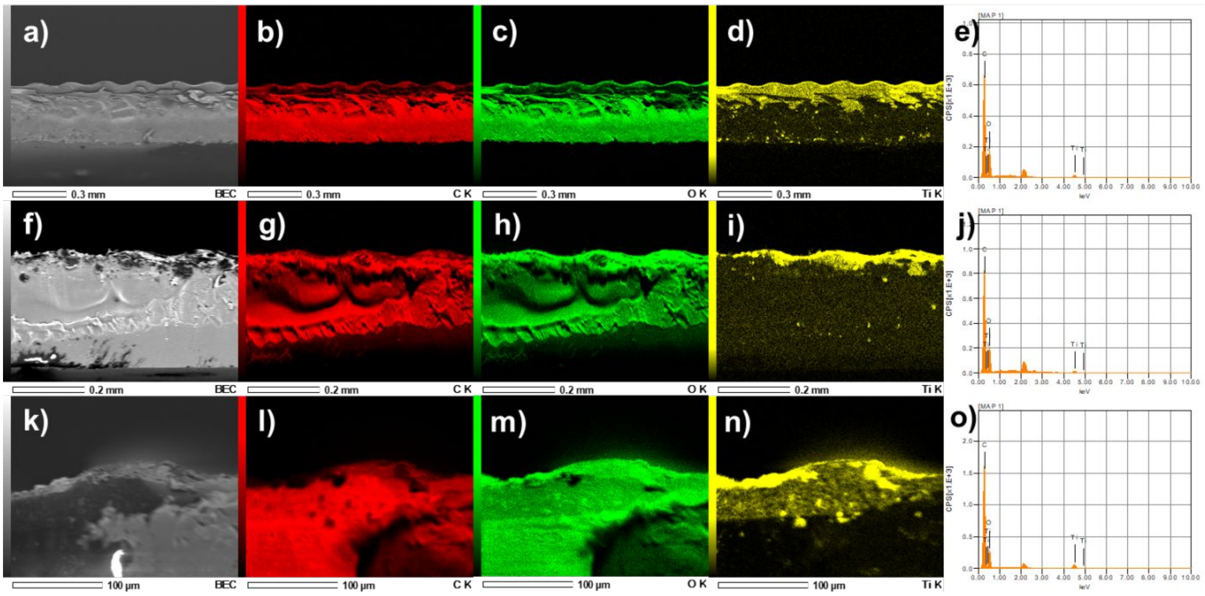

Figure S3. SEM images and EDS maps in cross–section of samples containing 8% nanoparticles obtained after exposure times of a)–e) 1 hour, f)–j) 4 hours, and k)–o) 24 hours. Corresponding spectra are also shown.

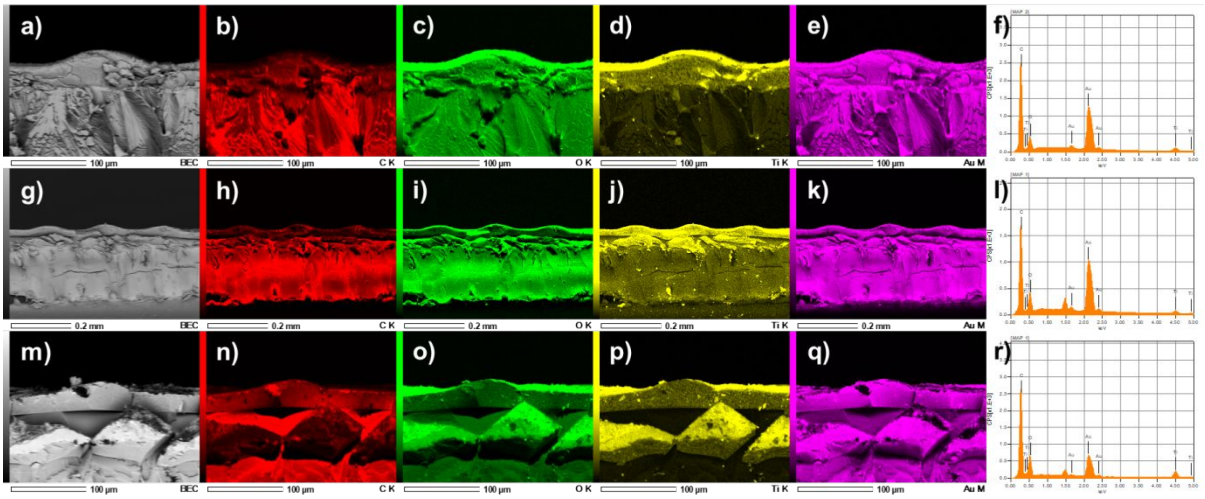

Figure S4. SEM images and EDS maps in cross–section of samples containing 16% nanoparticles obtained after exposure times of a)–f) 1 hour, g)–l) 4 hours, and m)–n) 24 hours. Corresponding spectra are also shown.

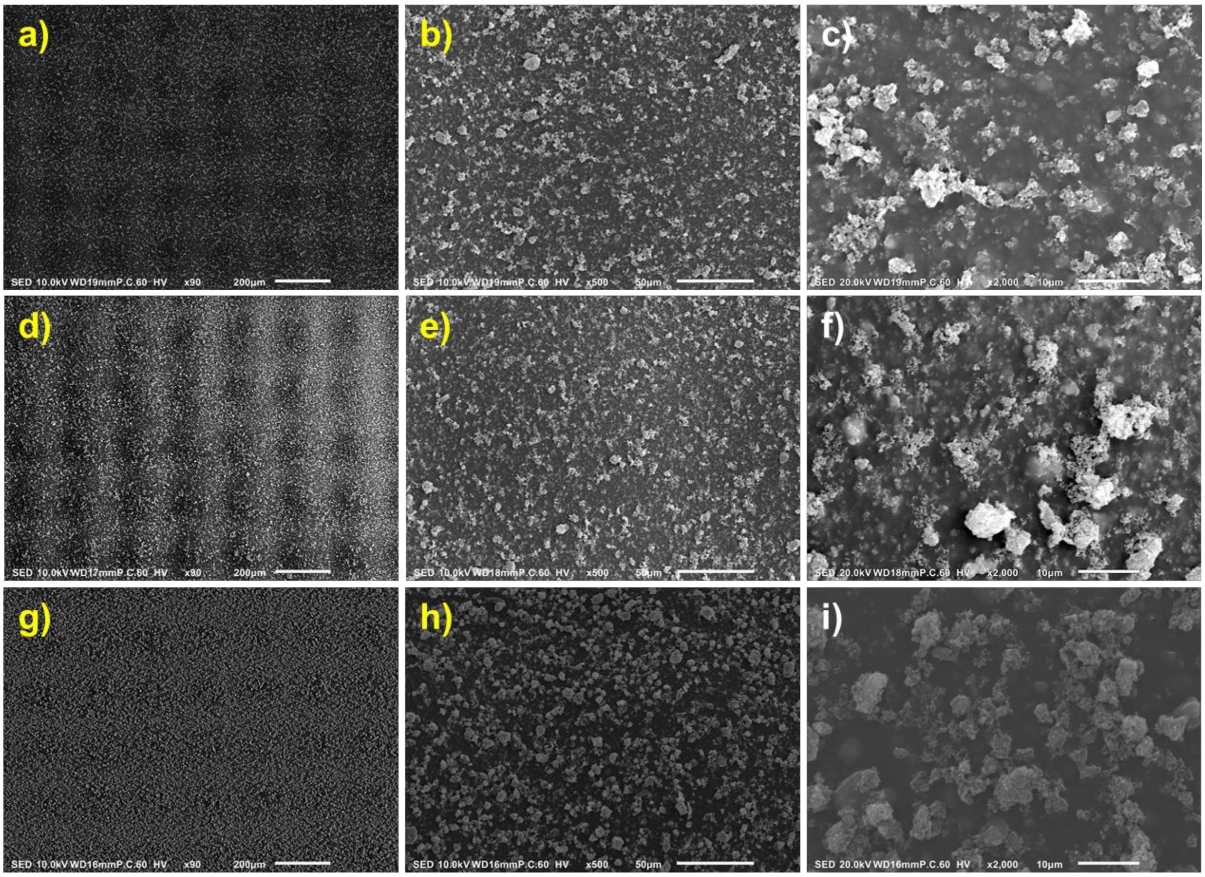

Figure S5. SEM images in top–down mode showing the top surfaces of samples containing 3% nanoparticles obtained after exposure times of a)–c) 1 hour, d)–f) 4 hours, and g)–i) 24 hours.

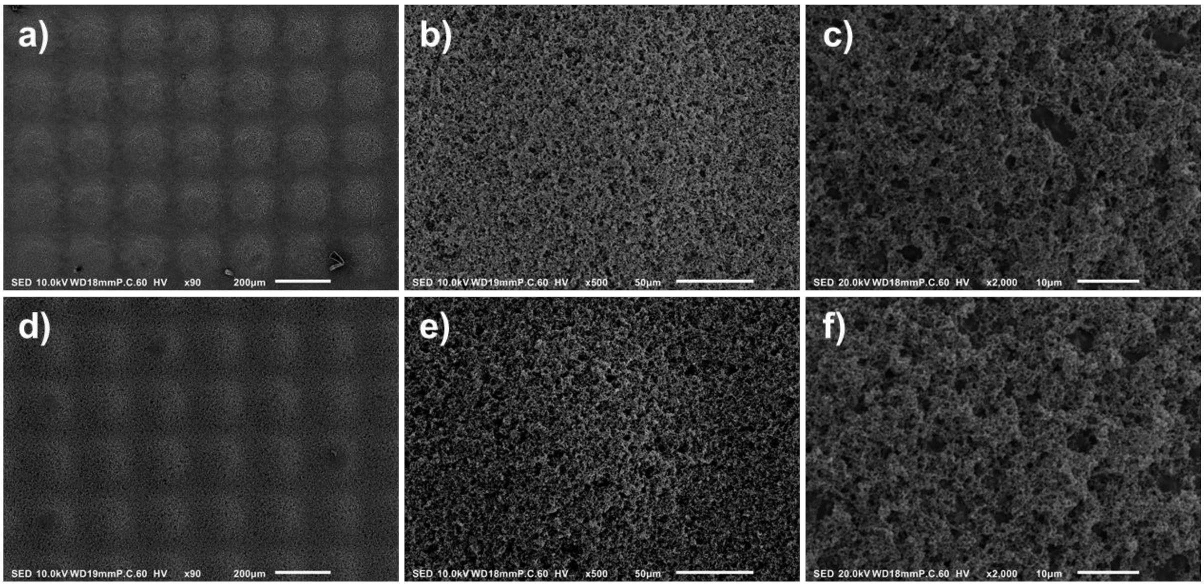

Figure S6. SEM images in top–down mode showing the top surfaces of samples containing 5% nanoparticles obtained after exposure times of a)–c) 1 hour, and d)–f) 4 hours.

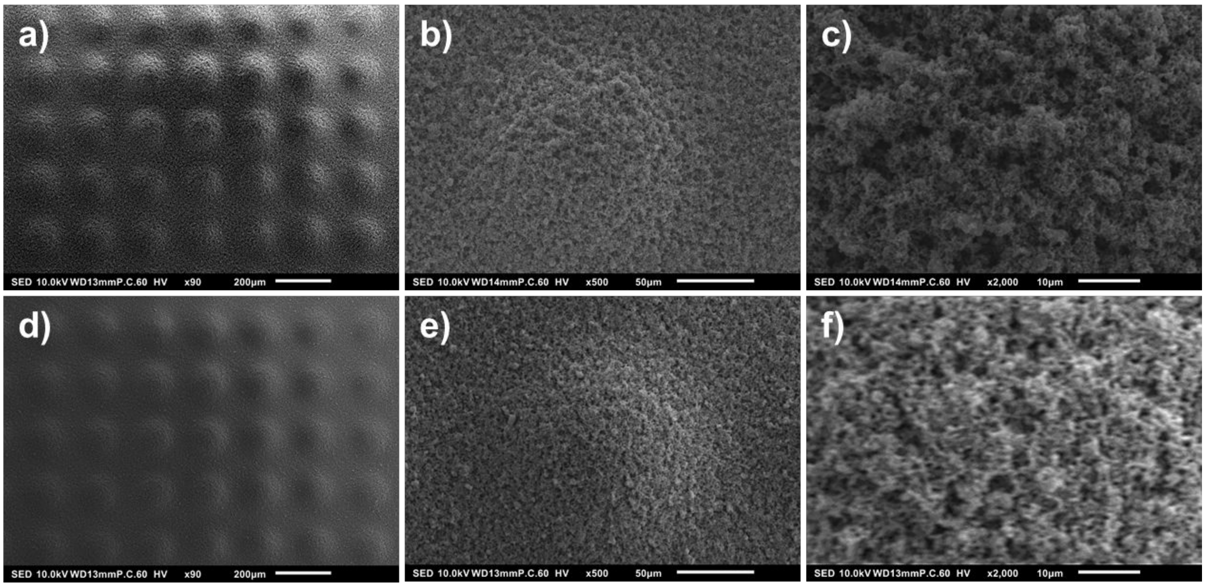

Figure S7. SEM images in top–down mode showing the top surfaces of samples containing 8% nanoparticles obtained after exposure times of a)–c) 1 hour, and d)–f) 4 hours.

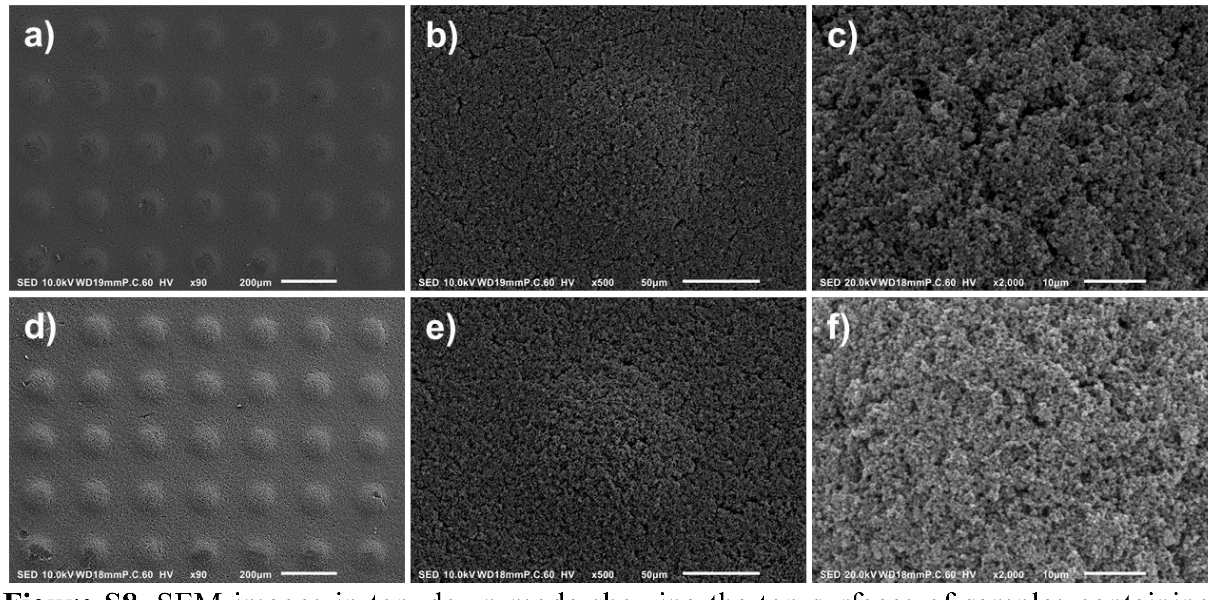

Figure S8. SEM images in top–down mode showing the top surfaces of samples containing 16% nanoparticles obtained after exposure times of a)–c) 1 hour, and d)–f) 4 hours.

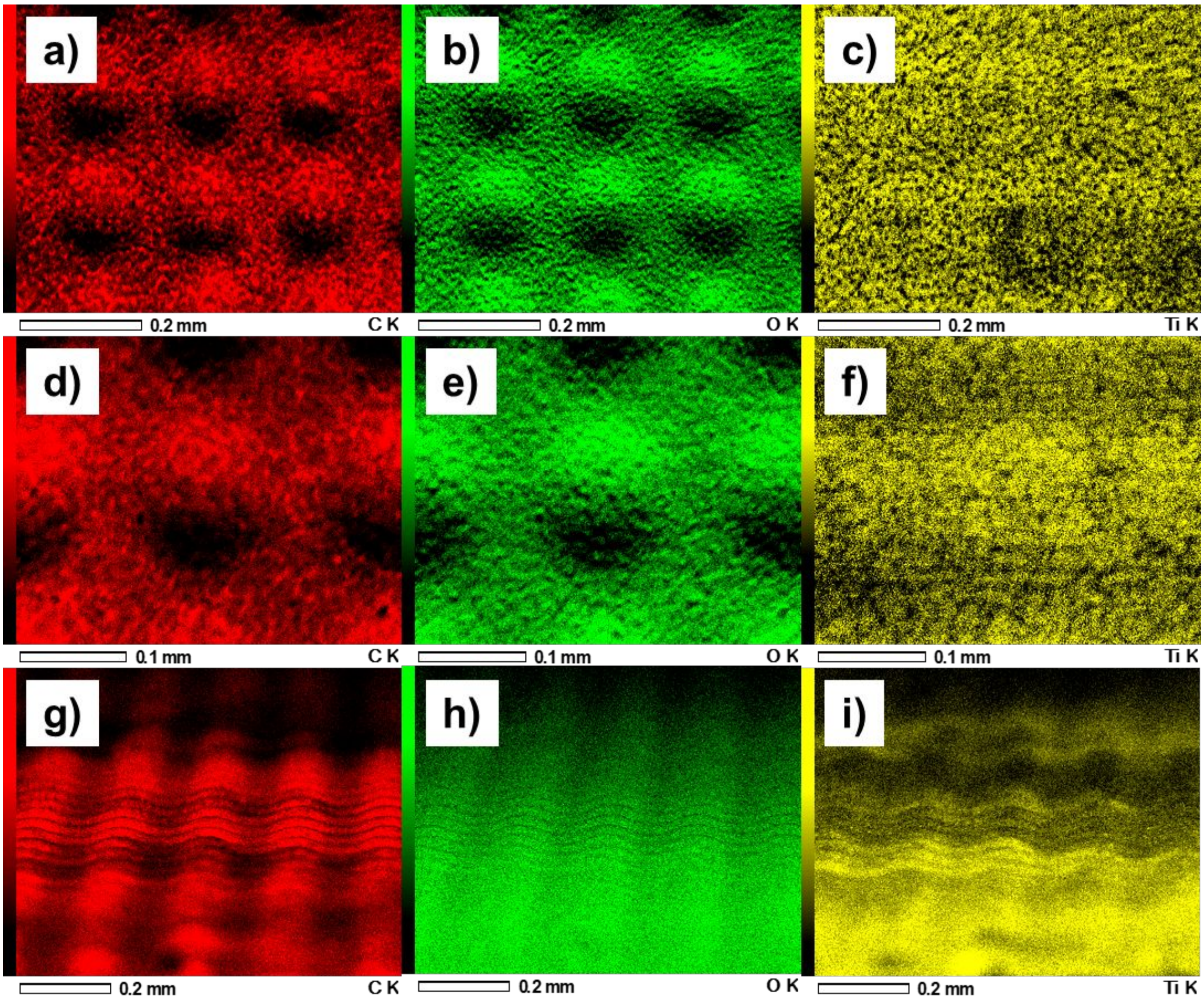

Figure S9. EDS maps in top–down mode showing the top surfaces of samples containing 8% nanoparticles obtained after exposure times of a)–c) 1 hour, d)–f) 4 hours, and g)–i) 24 hours (in perspective view).

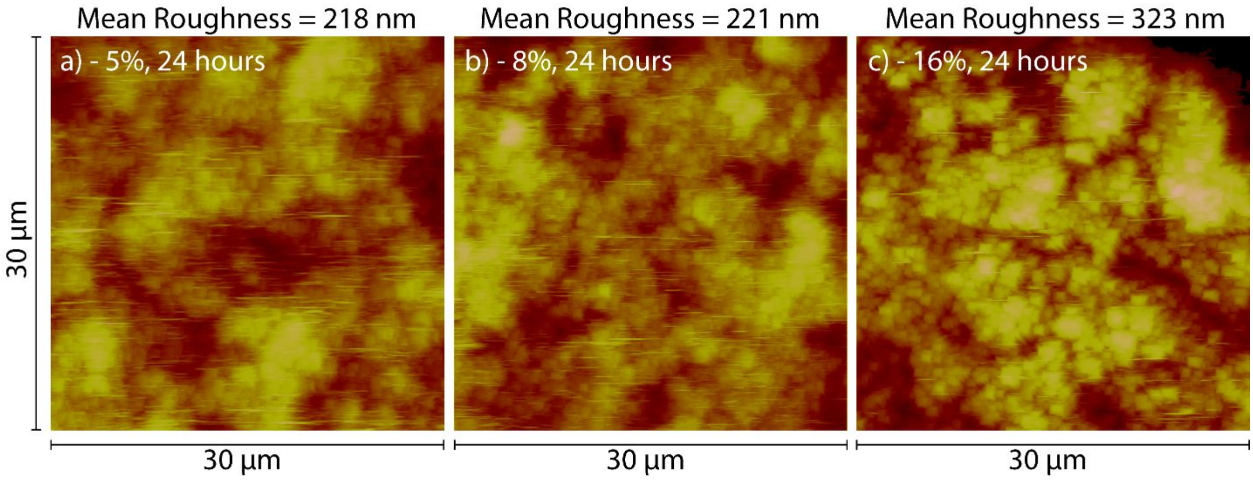

Figure S10. AFM height images of the superhydrophobic samples.

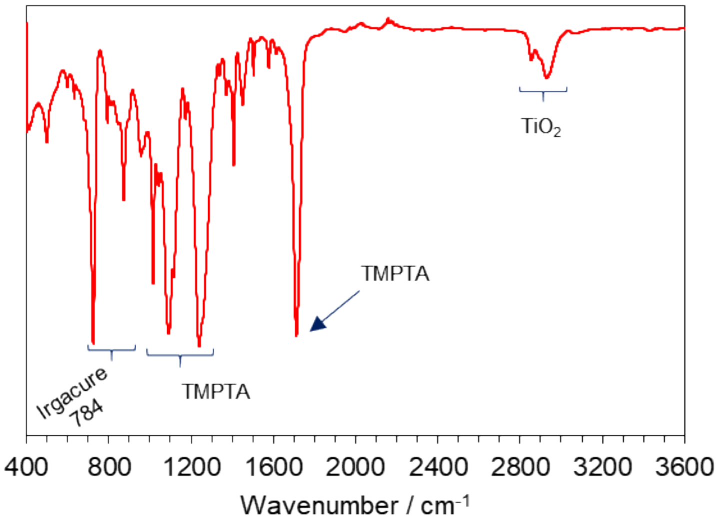

Figure S11. FTIR spectra of the sample of an 8%, 24 hours sample.

Table S1. Table of FTIR peak assignments

Peak Position [cm^{-1}]	Assignment	Component
3434	O–H stretching	TMPTA
2930	C–H stretching	TiO_2
2900	Likely C–H	TMPTA or TiO_2
2855	C–H stretching	TiO_2
2328	N/A	TiO_2
2119	Possibly R–N–C	Irgacure 784
1714	C=O stretching	TMPTA
1615	C=C stretching	TMPTA
1577	C=C aromatic or N–H	Irgacure 784
1504	C=C aromatic	Irgacure 784
1449	C–H methyl	TiO_2
1407	C–H	TMPTA
1370	Likely C–H	TiO_2
1321	N/A	N/A
1241	C–O or C–H	TMPTA
1172	C–O	TMPTA
1115	C–O	TMPTA
1091	N/A	TiO_2
1061	C–O	TMPTA
1015	Likely C–F	Irgacure 784
957	C–H vinyl	TMPTA
872	C–H aromatic	Irgacure 784
843	C–H vinyl	Irgacure 784
792	C–H aromatic	Irgacure 784
723	C–H aromatic	Irgacure 784
631	Likely C–F or TiO_2	Irgacure 784 or TiO_2
498	Likely C–F	Irgacure 784

Table S2. Summary of advancing contact angle (ACA), receding contact angle (RCA), and contact angle hysteresis (CAH) values for all nanoparticle weight fractions and irradiation times explored.

Nanoparticle Weight Content	Irradiation Time (hour)	ACA (°)	RCA (°)	CAH (°)
3%	1 hour	106.20	71.20	35.00
	4 hours	120.80	92.40	28.40
	24 hours	121.90	103.80	18.10
5%	1 hour	145.10	121.00	24.10
	4 hours	154.80	149.20	5.60
	24 hours	153.30	153.00	0.30
8%	1 hour	155.60	135.90	19.70
	4 hours	158.40	151.80	6.60
	24 hours	162.20	159.40	2.80
16%	1 hour	118.20	102.40	15.80
	4 hours	152.20	143.40	8.80
	24 hours	145.00	142.00	3.00

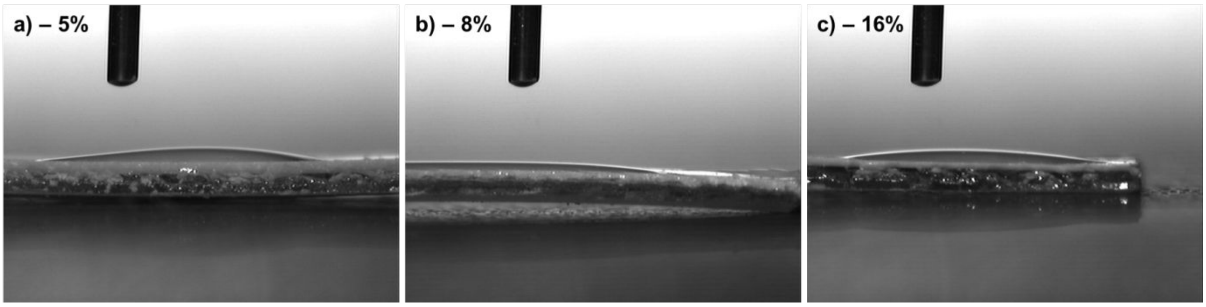

Figure S12. Static contact angle measurements taken on composite samples obtained without the use of a photomask.

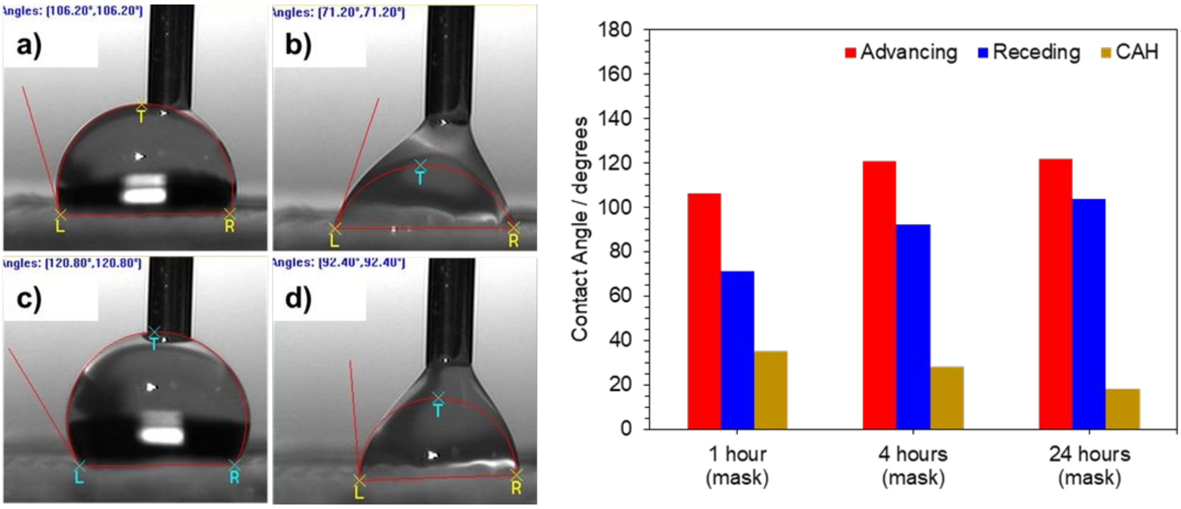

Figure S13. Advancing and receding contact angle measurements taken on samples containing 3% nanoparticles obtained after exposure times of a)–b) 1 hour, and c)–d) 4 hours. Bar plot e) summarizes the ACA, RCA and CAH values for these samples.

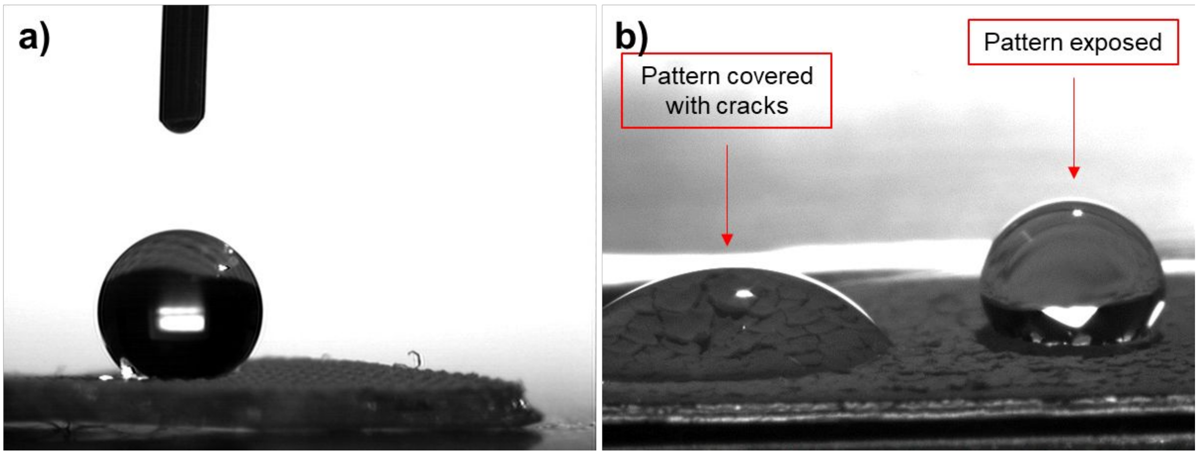

Figure S14. Digital photographs of a) a sessile water droplet placed on an 8%, 24 hours sample, taken in perspective view, and b) water droplets placed on two different regions of a 16%, 24 hours sample, wherein the cracked, nanoparticle–dense surface is visible in the droplet to the left, which leads to reduced anti–wetting properties.

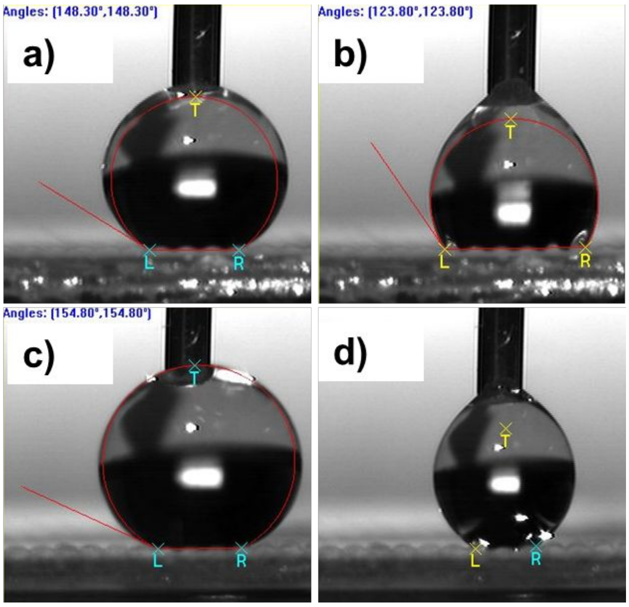

Figure S15. Advancing and receding contact angle measurements taken on samples containing 5% nanoparticles obtained after exposure times of a)–b) 1 hour, and c)–d) 4 hours.

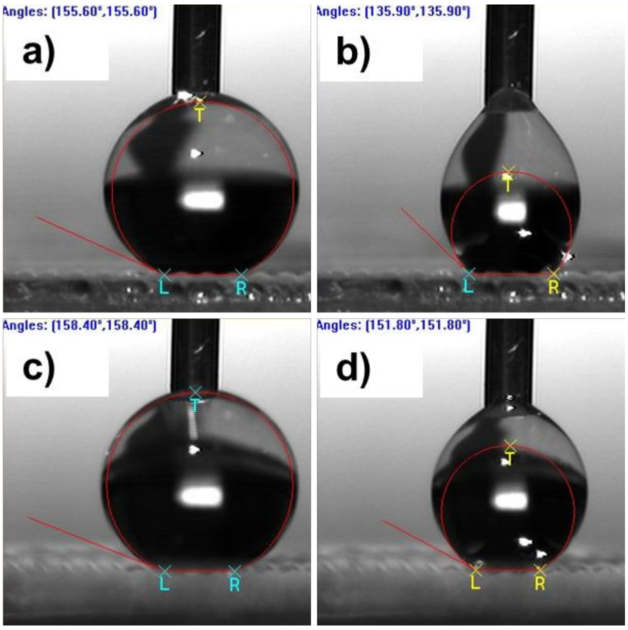

Figure S16. Advancing and receding contact angle measurements taken on samples containing 8% nanoparticles obtained after exposure times of a)–b) 1 hour, and c)–d) 4 hours.

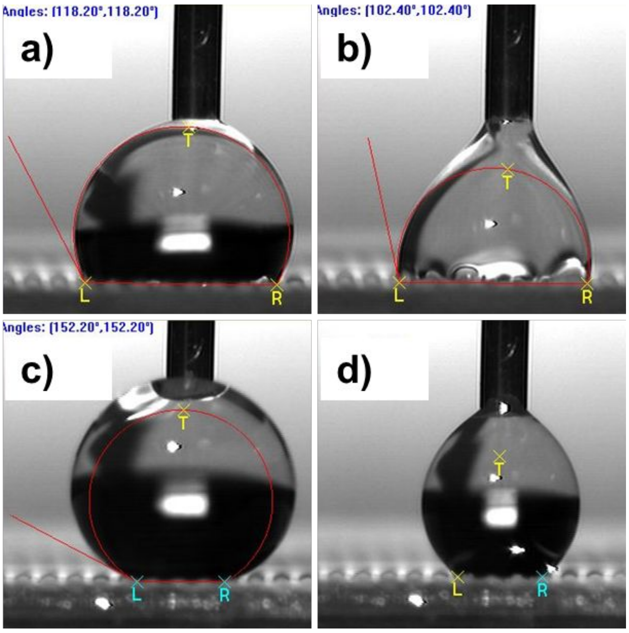

Figure S17. Advancing and receding contact angle measurements taken on samples containing 16% nanoparticles obtained after exposure times of a)–b) 1 hour, and c)–d) 4 hours.

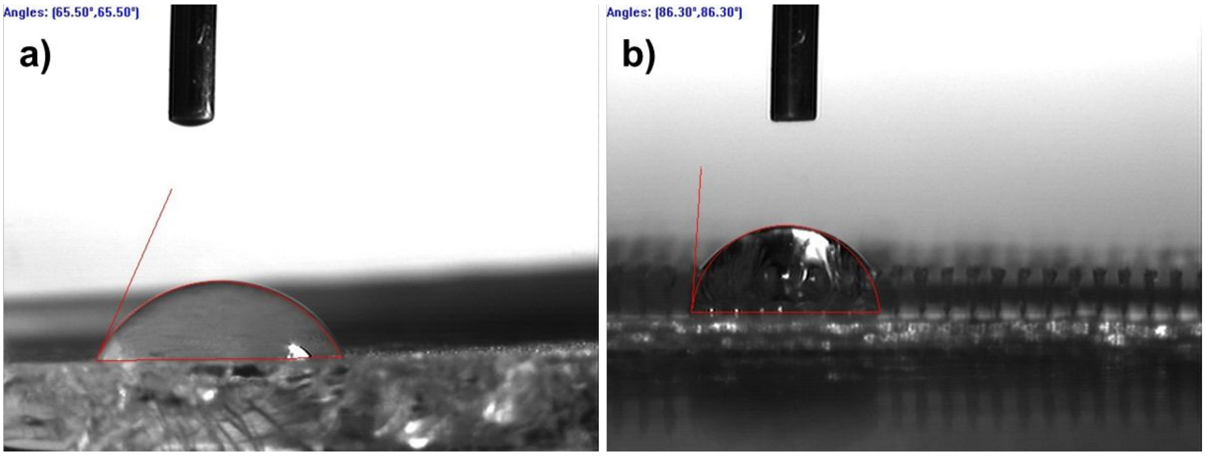

Figure S18. Static contact angle measurements taken on pure TMPTA samples with different surface textures – a) flat film, and b) pillars.

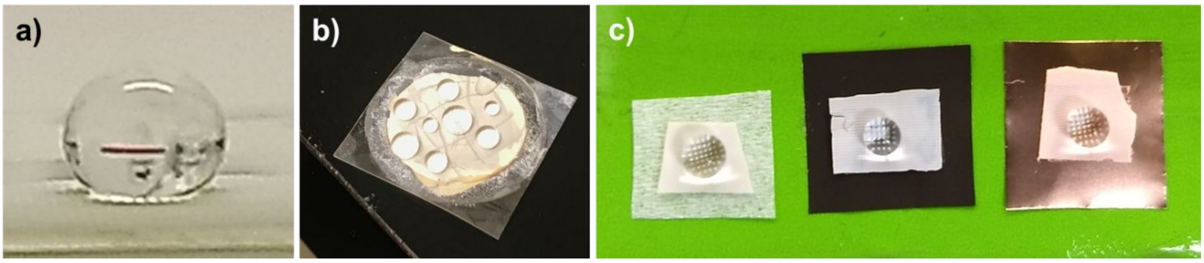

Figure S19. Digital photographs of a) a single sessile water droplet placed on the surface of a sample, b) several water droplets placed on the sample, and c) sessile water droplets placed on polymer composite samples attached to different substrates (top–down view), wherein the underlying surface texture is clearly visible through the droplets.

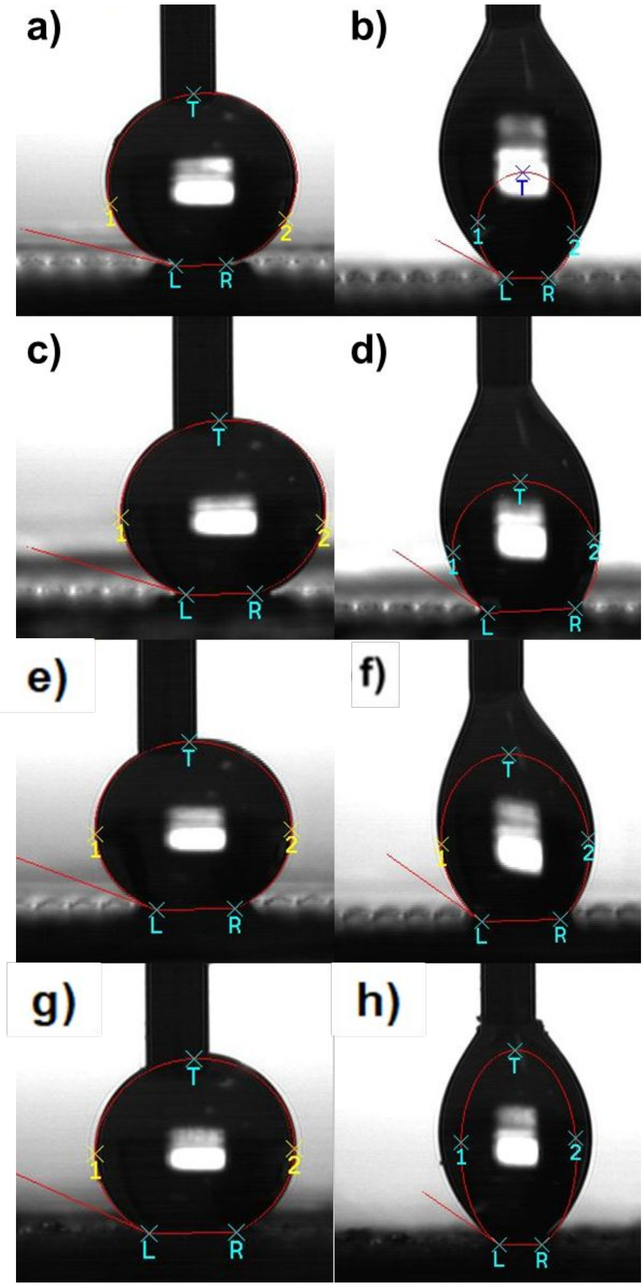

Figure S20. Advancing and receding contact angle measurements taken on samples containing 8% nanoparticles obtained after an exposure time of 24 hours, and post–mechanical testing as follows: a)–b) after peel 2, c)–d) after peel 3, e)–f) after peel 4, and g)–h) after abrasion 1.
